# Supplementary material for: Changes in Exercise Capacity and Ventricular Function in Arrhythmogenic Right Ventricular Cardiomyopathy: The Impact of Sports Restriction during Follow-Up
Source: J Clin Med. 2022 Feb 22;11(5):1150. doi: 10.3390/jcm11051150 (PMC8911196; doi:10.3390/jcm11051150)
Supplement: Supplementary file 1 [file jcm-11-01150-s001.zip › jcm-1566210-supplementary.pdf]

Supplemental Table S1. Exercise testing data.

	Adherent N = 37			Non-adherent N = 12		
	**Baseline**	**Follow-Up**	***p* Value**	**Baseline**	**Follow-Up**	***p* Value**
Max. workload (Watt)	164 ± 79	157 ± 73	0.394	213 ± 55	177 ± 53	0.012
Max. workload (% of predicted)	96 ± 36	94 ± 35	0.794	133 ± 25	116 ± 34	0.025
Max. heart rate (bpm)	146 ± 33	139 ± 28	0.151	157 ± 26	150 ± 28	0.319
Max. heart rate (% of predicted)	88 ± 19	84 ± 16	0.166	95 ± 12	91 ± 17	0.436
DP-factor [mean ± SD]	3.1 ± 0.9	3.1 ± 0.9	0.852	3.7 ± 0.8	3.1 ± 0.8	0.012

Supplemental Table S2. Echocardiographic data (available in=40).

	Adherent			Non-adherent		
	**Baseline**	**Follow-Up**	***p* Value**	**Baseline**	**Follow-Up**	***p* Value**
LVEF (%) [mean ± SD]	55 ± 10 (N=33)	55 [11.5] (N=32)	0.151	61 ± 6 (N=10)	54 ± 9 (N=10)	0.082
FAC (%)	33 ± 11 (N=34)	28 ± 10 (N=32)	0.794	34 ± 10 (N=11)	33 ± 6 (N=10)	0.192
RVOT PSAX (cm/m^2)	2.1 ± 0.6 (N=13)	2.0 ± 0.4 (N=22)	0.839	2.0 [0.2] (N=4)	2.0 ± 0.3 (N=5)	0.332
RVOT PLAX (cm/m^2)	2.1 ± 0.5 (N14)	2.0 ± 0.5 (N=20)	0.861	2.0 [0.2] (N=4)	2.0 [0.5] (N=5)	0.219
RVEDAi (cm/m^2)	15.0 ± 4.3 (N=32)	14.6 [3.9] (N=31)	0.852	15.8 ± 3.6 (N=11)	16.7 [2.2] (N=10)	0.969

Left ventricular ejection fraction (LVEF), fractional area change (FAC), right ventricular outflow tract (RVOT), parasternal short axis (PSAX), parasternal long axis (PLAX), right ventricular end-diastolic area indexed (RVEDAi)

Supplemental Table S3. 24 h Holter ECG data.

	Baseline		Follow-up	
	**Non-adherent N = 11**	**Adherent N = 31**	**Non-adherent N = 7**	**Adherent N = 20**
**< 500 PVC**	3 (27%)	12 (39%)	4 (57%)	8 (40%)
**≥ 500 PVC**	8 (73%)	19 (61%)	3 (43%)	12 (60%)
**Overall PVC burden** [median [IQR]]	1232 [2372]	1531 [2414]	261 [584]	705 [1635]

PVC: premature ventricular contraction.
